# Kinetic of Light Transmission during Setting and Aging of Modern Flowable Bulk-Fill Composites

**DOI:** 10.3390/ma17174292

**Published:** 2024-08-30

**Authors:** Nicoleta Ilie, Christof Högg

**Affiliations:** Department of Conservative Dentistry and Periodontology, University Hospital, Ludwig Maximilians University, Goethestr. 70, D-80336 Munich, Germany; christof.hoegg@med.uni-muenchen.de

**Keywords:** bulk-fill composite, light transmittance, hardness, modulus of elasticity, creep, aging

## Abstract

The current development of dental materials aims to improve their properties and expand their clinical application. New flowable bulk-fill composites have been released which, unlike what was previously common in this material category, are intended to be used alone and without a top layer, in various cavities. The study compares their kinetic of light transmission during monomer-to-polymer conversion on a laboratory-grade spectrometer, as well as their elastoplastic and aging behavior under simulated clinical conditions. Major differences in the kinetic of light transmission was observed, which is related to the degree of mismatch between the refractive indices of filler and polymer matrix during polymerization and/or the type of initiator used. Compared to the literature data, the kinetic of light transmission do not always correlate with the kinetic of functional group conversion, and therefore should not be used to assess polymerization quality or to determine an appropriate exposure time. Furthermore, the initial mechanical properties are directly related to the volumetric amount of filler, but degradation during aging must be considered as a multifactorial event.

## 1. Introduction

Bulk-fill application technique in 4–5 mm increments of polymer-based composites is now widely used in dentistry [1] for faster [2] deep-cavity restoration. Apart from the time and implicit cost savings, this technique is well suited for patients with lower compliance and for children or elderly patients.

The low-viscosity, so-called flowable variants of bulk-fill composites are enjoying increasing popularity, as they are easier to handle compared to the highly viscous formulations and have good flow properties that allow the quick filling of large gaps but also the infiltration of very narrow, deep clefts. Reduced viscosity and tailored flowability are both controlled by the size and amount of filler [3]; consequently, flowable bulk-fill composites receive a consistently lower amount of filler, which has a negative impact on several mechanical properties and, in particular, the elastic modulus [4,5] and wear resistance [6]. In vitro it was shown that the lower elastic modulus of flowable bulk-fill composites after aging of Class II restorations has no influence on the marginal gap in dentin, but clearly has a negative impact on the marginal gap in enamel [7]. The lower amount of filler and the associated higher polymer content also result in greater polymerization shrinkage, which manifests itself in a restricted cavity through increased shrinkage stress and higher imperfect margin [8,9]. Both deciduous and permanent thermally aged molar restorations performed in vitro are inferior in terms of marginal integrity when restored with flowable bulk-fill composites than with high-viscosity bulk-fill or traditional hybrid composites [10]. However, such results must be interpreted in the context of the study design and parameters such as the configuration factor (C-factor), i.e., the ratio of bound to unbound areas in a cavity, must be taken into account [11]. In contrast to the studies already mentioned, a recent study found no differences in initial gap formation between flowable bulk-fill materials and conventional materials, but rather a superiority of the former after thermal aging [12]. The data on the behavior of this material category thus remain heterogeneous.

A bulk-fill technique involves the placement of a large, up to 4–5 mm thick, flowable bulk-fill increment that is ultimately covered with a conventional, high-viscosity composite, or the placement of a high-viscosity bulk-fill composite in one 4–5 mm increment. The clinical success of such restorations has been proven in the past [13] and is confirmed by current studies [13]. These demonstrate that the use of a flowable bulk-fill composite in vivo in a 4 mm increment covered by a conventional composite in Class II restorations has good clinical performance and is similar to that of incremental cavity reconstruction with the conventional composite only, until 24 months [14]. However, the initial definition of a bulk-fill technique appears to be misinterpreted in recent perception, as flowable bulk-fill composites have been used clinically in deep cavities without additional capping. Flowable bulk-fill composits applied in a single increment in 138 up to 4 mm deep Class III restorations, appear to perform well in clinical use for up to one year of observation [15]. Despite the ongoing development of novel materials and the goal of versatility and universal application, it is recommended that caution be exercised when filling large posterior cavities with flowable bulk-fill composites [5]. These concerns are primarily based on the fact that flexural strength alone cannot be used as a basis for deciding on the use of composite materials. Although the flexural strength of flowable bulk-fill composites measured 24 h after polymerization is well over 80 MPa, which represents the clinically acceptable lower limit of strength for the use of a restorative material in the posterior region [16], this value is achieved due to their high flexibility and is not supported by a suitable high elastic modulus [5]. In addition, a low-filled material with a higher polymer content is expected to degrade [17] more rapidly under clinical conditions, further reducing the mechanical parameters over time [18] and affecting the durability of a restoration [19]. Aging represent a difficult challenge in all composite restorations [17], as their composition involves polymers with hydrolysable bonds such as esters, ethers, urethanes, and amides that can be cleaved by hydrolysis [20]. While the potential for degradation is inherent in the nature of the materials, degradation occurs in composite materials despite varying vinyl acrylate compositions [21].

Concerns about sufficient polymerization of bulk-fill materials at depth appear to have been addressed, as such materials have evolved into mature, complex materials incorporating all modern advances in materials science. In addition to the use of nanotechnology [22] and bioactivity for the fillers [22,23], the monomer matrix uses new polymerization mechanisms such as RAFT (Reversible Addition–Fragmentation Chain-Transfer Polymerization) [24], new polymer matrices [25] or photo-initiator systems [26]. As the development of novel materials advances rapidly, their thorough characterization and comparison in laboratory studies are essential to understand their clinically potential effects. The aim of the present study was therefore to offer a comparison of the kinetic of light transmission during setting in new and established flowable bulk-fill materials and to quantify their aging behavior under clinically simulated conditions up to 6 months.

The null hypotheses which are tested state that modern flowable bulk-fill composite formulations behaves similarly with respect to (a) the kinetic of light transmission during setting; (b) elasto-plastic mechanical parameters, and (c) aging behavior.

## 2. Materials and Methods

### 2.1. Materials

Three modern flowable bulk-fill RBCs (Table 1) were compared by evaluating the light transmittance during light curing in real-time. In addition, a range of micro-mechanical parameters were recorded to determine elastoplastic and viscoelastic material behavior up to an aging duration of 6 months. The light-curing unit (LCU) used for polymerization (Coltolux^®^ LED, Coltene Whaledent Inc., Cuyahoga Falls, OH, USA) was employed for 20 s for each material.

### 2.2. Methods

Light transmittance during the 20-s curing process was monitored in real time with 16 recordings per second. In addition, the mechanical material behavior was determined by a series of parameters collected using an instrumented indentation test 24 h post-polymerization as well as after 6 months of aging under clinically simulated conditions.

A. Real-time light transmission during light exposure

The light transmission was determined in real time during the whole exposure duration, which was 20 s in each material, on a laboratory-grade USB4000 spectrometer (MARC (Managing Accurate Resin Curing) System, Blue light Analytics Inc., Halifax, NS, Canada) referenced by the National Institute of Standards and Technology (NIST). The spectrometer uses a 3648-element Toshiba linear Charge-coupled Device (CCD) array detector and high-speed electronics (Ocean optic, Largo, FL, USA). It was calibrated by means of an Ocean Optics’ NIST-traceable light source (300–1050 nm) and employs a CC3-UV Cosine Corrector (Ocean optic, Largo, FL, USA) to collect radiation over a 180° field of view. In this way, the effects of optical interference associated with the geometry of the light collection scan are mitigated.

The incident irradiance was measured by placing the LCU in direct contact, centered and perpendicular to the spectrophotometer’s sensor, which has a diameter of 3.9 mm. It amounts to the irradiance striking the sample surface as the composite cures. Next, to measure the transmitted irradiance, a 2 mm-thick sample of uncured composite paste, enclosed by a circular white plastic mold (inner diameter 6 mm), was placed between the LCU and the sensor. Measurement of transmitted irradiance starts with switching on the LCU, and this was monitored during the 20 s of light exposure. Irradiances and spectral distribution in a wavelength range of 360–540 nm were recorded individually at a rate of 16 recordings/s. The sensor was triggered at 20 mW.

B. Instrumented indentation test (IIT): quasi-static approach (ISO 14577 [27]) to determine micro-mechanical properties

Paralelipipedic samples (length = 16 mm, width = 2 mm, height = 2 mm) were prepared in white, polyoxymethylene, molds according to the recommendations described in ISO 4049 [16]. All samples (*n* = 10 for each material) were exposed to light for 20 s, with the overlap of the exposed areas being less than 1 mm. The exposure was from both sides. After removal from the mold, the samples were first stored in distilled water at 37 °C for 24 h. Half (*n* = 5) of these for each material were subsequently tested and the other half were immersed in artificial saliva at 37 °C for 6 months, with the immersion medium renewed weekly. Prior to measurement, each sample was fixed to a glass slide, mounted in an automatic grinding machine (EXAKT 400CS Micro Grinding System EXAKT Technologies, Inc., Oklahoma City, OK, USA), wet-ground with silicon carbide sand paper (grit size p2500 and p4000, LECO Corporation, St. Joseph, MI, USA) and polished with a diamond suspension (mean grain size: 1 µm). The measurements were carried out with an automatic micro-hardness indenter (FISCHERSCOPE^®^ HM2000, Helmut Fischer, Sindelfingen, Germany), in a quasi-static approach. For this purpose, indentation depth and force were monitored simultaneously during an indentation cycle, which includes an increase in indentation force within 20 s from 0.4 mN to 1000 mN, a holding time of 5 s at maximum force, and a subsequent reduction in force within 20 s at a constant speed. Change in indentation depth recorded during holding time served as a measure for the material’s creep (Cr). The indentation modulus (E_IT_) was calculated from the slope of the tangent of the indentation-depth curve at maximum force. Indentation creates an impression with projected indenter contact area (A_c_) determined from the force-indentation depth curve, considering the indenter correction based on the Oliver and Pharr model which is described in ISO 14577 [27]. Sapphire and quartz glass were used to calibrate the indenter-area function. Corrections obtained from the tip calibration were then used for further computational data analysis. The indentation hardness (H_IT_ = F_max_/A_c_) is a measure of the resistance to plastic deformation and was converted to the more familiar Vickers hardness (HV = 0.0945 × H_IT_ [27]). The universal hardness (or Martens hardness = F/A_s_(h)) was calculated by dividing the test load by the surface area of the indentation under the applied test load (As), giving a characterization of both plastic and elastic deformation.

C. Microstructural characterization

Scanning electron microscopy (SEM, Zeiss Supra 55 V P, Carl Zeiss AG, Oberkochen, Germany) was used to characterize the microstructure of the analyzed materials, and was operated in electron backscatter diffraction mode. Analyzed samples (*n* = 3) were cured and wet-processed as described above.

### 2.3. Statistical Analyses

The distribution of the variables was tested using the Shapiro–Wilk method. Following this, a parametric approach could be used. Multifactor analysis of variance was applied to compare the parameters of interest (Martens and Vickers hardness; creep; indentation modulus; and light transmittance). The results were compared using the confidence interval, one- and multiple-way analysis of variance (ANOVA) and Tukey’s honestly significant difference (HSD) *post-hoc* test using an alpha risk set at 5%. The effect of the main parameters and their combinations was assessed by a multivariate analysis (general linear model) and partial eta-squared statistic (SPSS Inc., Version 29.0, Chicago, IL, USA).

## 3. Results

### 3.1. Light Transmittance

**Figure 1:**
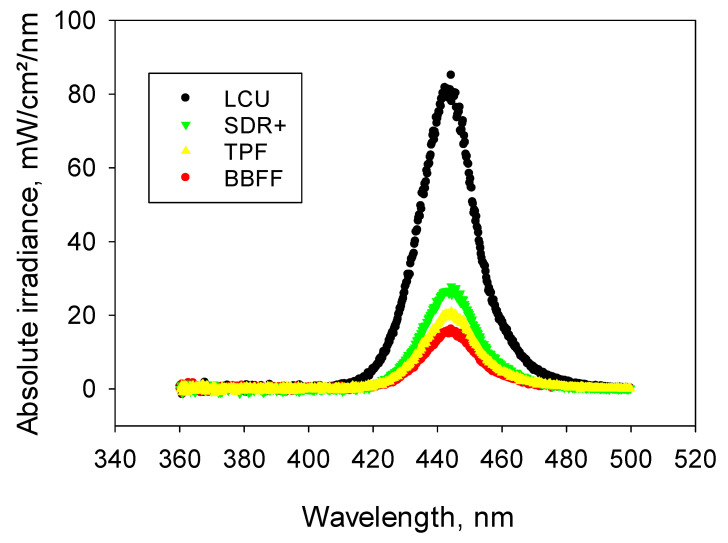
Spectral distribution of the LCU and light transmitted through a 2 mm increment of the analyzed composite materials.

Incident light amounted (1928.2 ± 5.1) mW/cm^2^ and the radiant exposure at an exposure time of 20 s was (38.7 ± 0.1) J/cm^2^. The employed LCU was a blue LED (light-emitting diode) device, with a spectral distribution in the blue wavelength ranging from 410 nm to 490 nm and a peak at 444.2 nm. The LCU reaches the maximum value 0.3 s after switching on, and remains constant thereafter. When passing through a 2 mm composite increment, the light is strongly attenuated for all wavelengths, with the most translucent material for all wavelengths being SDR+, followed by TPF and then BBFF. The spectral distribution of the blue LED LCU and the light transmitted through the analyzed materials can be depicted in Figure 1.

**Figure 2:**
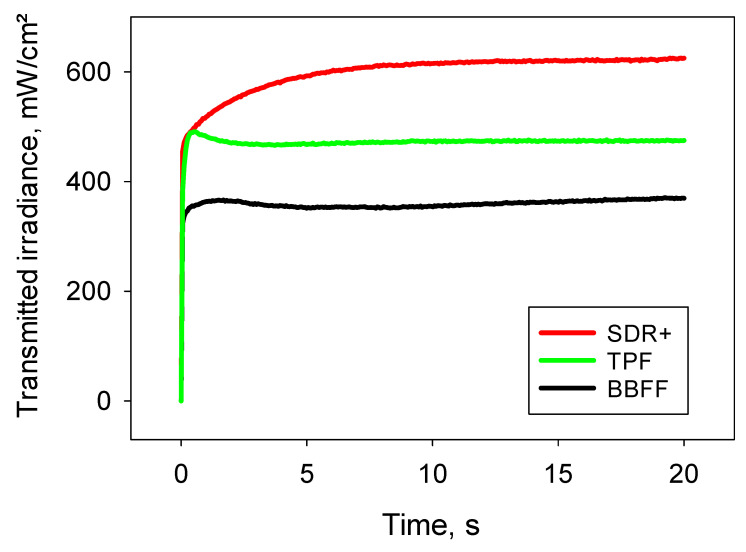
Real-time light transmission during the 20 s exposure protocol.

Apart from the spectral distribution, the analyzed materials differ significantly in the variation in the transmitted light during light exposure. The transmitted irradiance for SDR+ increases continuously during polymerization for up to 5–6 s of light exposure and then reaches a plateau at high values. The variation in transmitted irradiance with exposure time had a different shape for TPF, as it reached the highest value shortly after the start of exposure, decreased slightly as exposure progressed, and plateaued at a much lower level than SDR+ after 2–3 s of light exposure. In contrast, BBFF shows the smaller difference between the uncured monomer paste (first measured point by starting the LCU) and the forming polymer, and the curve plateaued fast and at the lowest light-transmittance value within the analyzed materials (Figure 2).

The above presented change in light transmittance during polymerization was then described for each material by an exponential sum function, as outlined in the equation below:

$$y=a\left(1-e^{-bx}\right)+c\left(1-e^{-dx}\right)\;$$

where the parameters a, b, c, d were the modulation factors of the exponential function to optimize the exponential function on the measured curve plotted on a transmitted irradiance versus the time curve. In this equation, the parameters a and b should characterize the initial part of the light transmittance, which includes the monomer-to-polymer conversion in the gel phase (=the Trommsdorff–Norrish effect), along with the photo-initiator consumption, while parameters c and d should describe the further course of the polymerization process when the reaction solution turns into a gel-like state and the reaction rate decreases (glass effect).

A very high correlation factor of R^2^ > 0.96 was calculated for all materials, while the modulation factors with their standard error can be depicted in Table 2. The 95% confidence interval can be then be assessed from the parameter value from which (1.96 × standard error) is added and subtracted. Significance occurs when the confidence intervals do not overlap.

For all materials, the fastest change in light transmittance occurred in the initial part of the curve and is characterized by parameters a and b. These changes were faster for SDR+, followed by BBFF and then TPF (descending order of parameter b, Table 2). The velocity of variation is then consistently lower in all materials (small d-parameter), while the changes show increased light transmittance (positive c-parameter), which was high for SDR+ (high c-parameter) and low for BBFF, while the light transmittance decreases with TPF (negative c-parameter), meaning the material becomes more opaque.

**Figure 3:**
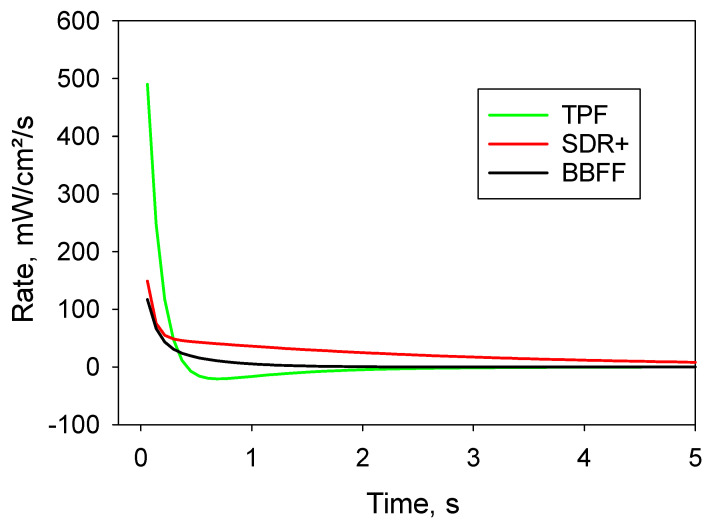
Rate of transmitted light during monomer–polymer conversion.

In addition, the rate of variation in time of transmitted irradiance during curing was calculated, and can be depicted in Figure 3 for the first 5 s of irradiation. The highest initial rate was observed for TPF, while it was four-times lower and comparable for SDR+ and BBFF.

For TPF, the rate drops to zero within the first 0.45 s of light exposure and then becomes negative up to 2 s of cure, meaning the material becomes more opaque. The rate then levels off towards zero, which means that no further change in light transmission has occurred. In contrast, SDR+ starts with a much lower initial rate, decreasing rapidly up to 0.37 s of light exposure and then steadily decreasing, but at a slower rate, to reach a value close to zero only after 4.5 s. As for BBFF, the curve shows a similar two-stage decrease velocity, with the first being faster, up to 0.31 s, and the second slower, with a rate compared to SDR+, which reaches the level of zero, meaning no significant further changes in light transmittance, after 2 s of light-exposure.

**Figure 4:**
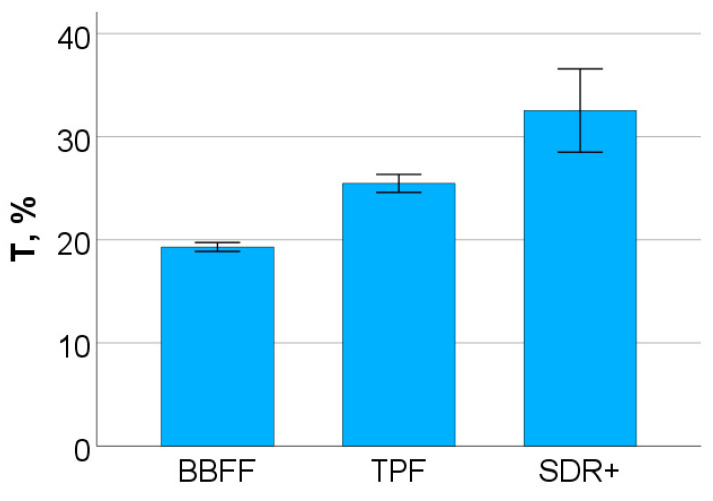
Light transmittance (T%) at the end of the exposure protocol.

The exposure protocol ended after 20 s for all materials, to ensure similar curing conditions. The light transmittance T, defined as the ratio of the radiant flux (irradiance) transmitted through a sample to the radiant flux incident on the sample, was highest for SDR+, followed by TPF and then BBFF (*p* < 0.001; Figure 4). One needs to point out that, even for the most translucent material, the loss of incident light when passing through a 2 mm composite layer is very high, approaching 70%.

### 3.2. Instrumented Indentation Test (IIT): Quasi-Static Analysis Mode

The micromechanical parameters were assessed 24 h after polymerization and after aging the samples in artificial saliva for 6 months. A significant (*p* < 0.001) decrease (increase in creep) with aging was observed for all materials, with the extent of the change being material- and parameter-dependent.

The measured data were quantified by multifactorial analysis and the effect size of the main parameters—filler volume% and aging—as well as their interaction on the measured properties being calculated. The effect of filler volume% and aging was all over significant (*p* < 0.001). The effect strength was quantified by the partial eta-squared values, and is summarized in Table 3. The filler volume percentage showed the strongest significant influence on all measured parameters (higher partial eta-squared values), except for creep. Although the aging effect is smaller than the filler effect, it can still be classified as strong, while the binary-interaction effects of the main parameters were significant and moderate.

**Figure 5:**
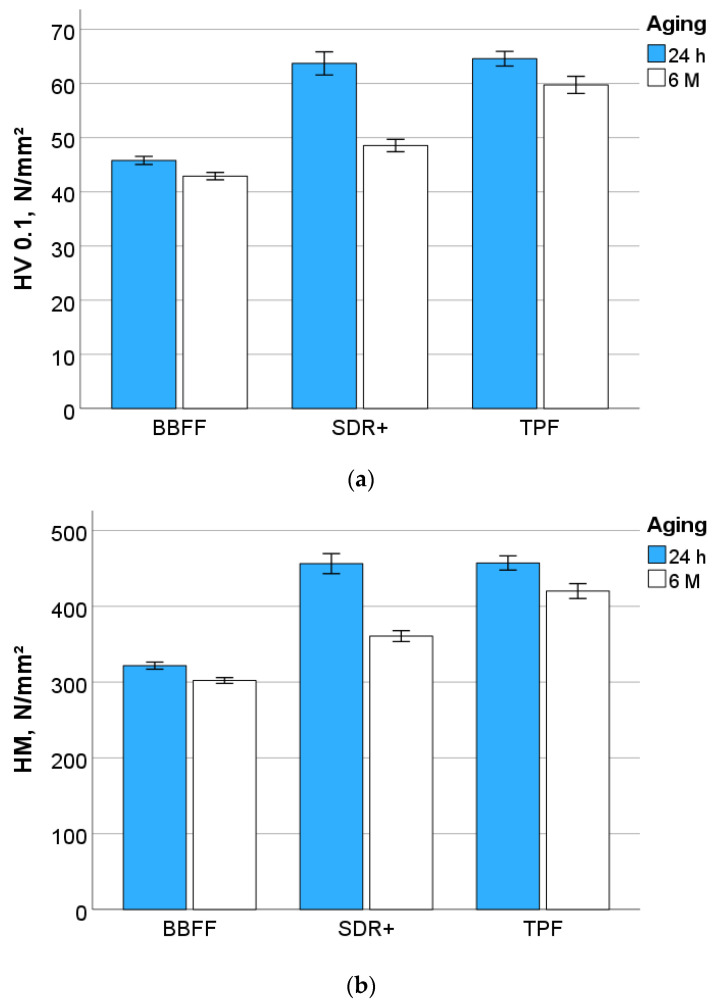

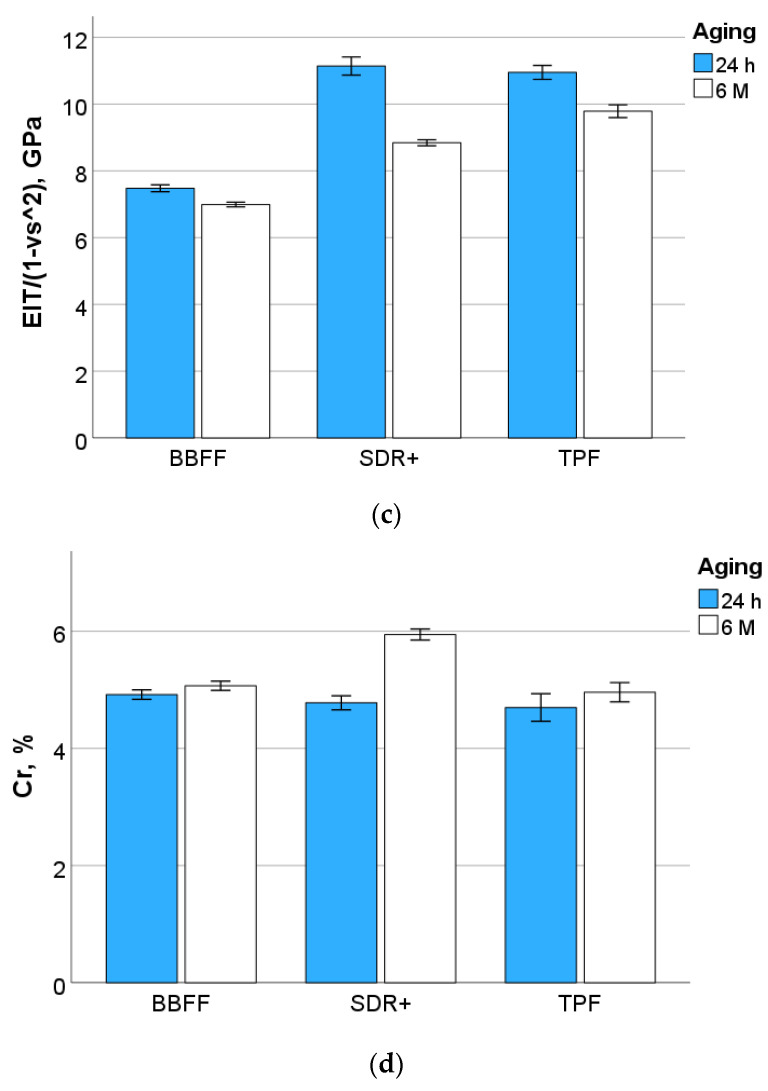
Outcome of the instrumented indentation test IIT (quasi-static mode): (**a**) Vickers hardness, (**b**) Martens hardness, (**c**) indentation modulus, and (**d**) creep.

As an exponent of plastic deformation, Vickers hardness (Figure 5a) reveals similar and the significantly highest values for TPF and SDR+ (*p* = 0.665) compared to BBFF (*p* < 0.001) 24 h after light exposure. For all materials, aging causes a significant decrease in HV, which was more pronounced for SDR+, followed by TPF and then BBFF. As a result, the differences between the materials become significant after aging in the material sequence BBFF < SDR+ < TPF (*p* < 0.001).

The material sequence for HM (Figure 5b) is related to that presented above, showing similar and significantly highest values for TPF and SDR+ (*p* = 0.991) compared to BBFF (*p* < 0.001) 24 h after curing, as well as a similar variation and a statistical relationship between materials with aging. On an analogous path, the indentation modulus 24 h post-polymerization was similar for TPF and SDR+ (*p* = 0.335) and significantly higher compared to BBFF (*p* < 0.001). The faster decline with aging in SDR+ differentiates the materials in the same sequence with respect to the hardness values (*p* < 0.001) (Figure 5c). In contrast, all three materials behave statistically similarly (*p* = 0.103, 0.389, 0.723) 24 h post-polymerization with regard to creep (Figure 5d), while aging induced a higher value in SDR+ compared to BBFF and TPF (*p* < 0.001), which behaved similarly (*p* = 0.344).

### 3.3. Scanning Electron Microscopy (SEM)

**Figure 6:**
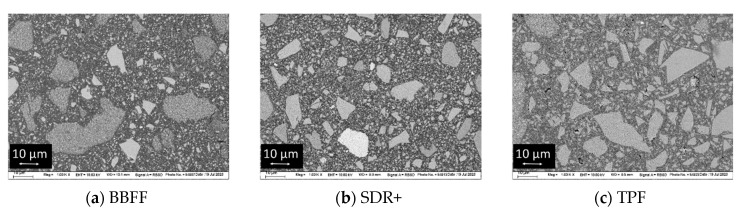
Structural appearance of the filler systems at 1000× magnification, measured in electron backscatter diffraction mode using scanning-electron microscopy.

SEM images of the analyzed materials can be depicted in Figure 6. The electron backscatter diffraction mode was chosen for investigation to enable discrimination between fillers with different chemical compositions, as higher atomic-order elements in the filler determine a brighter appearance. In fact, two different types of fillers can be distinguished: compact fillers with irregular shape in all materials, together with pre-polymerized fillers (PPFs) in BBFF and TPF.

## 4. Discussion

Flowable bulk-fill composites have experienced over the years a significant expansion of their clinical indication. Initially, they were recommended for filling narrow and deep gaps due to their improved flow performance and ability to cure in 4–5 mm. Subsequently, flowable bulk-fill composites were indicated for the reconstruction of ever-larger cavities, but only in conjunction with an additional top-layer composite with a higher filler content. Recently, according to the manufacturer, modern formulations require neither a liner nor an additional top layer to ensure stability and longevity, even when placed in stress-bearing areas.

While adequate curing in 4–5 mm increments has been demonstrated for all materials [5] and is tested extensively prior to market launch, there remains a need for discussion regarding the sufficiency of mechanical properties to withstand areas exposed to high mechanical stress, along with the way materials alter with aging.

In designing a light-curing composite, good light transmittance in depth is a prerequisite for proper monomer-to-polymer conversion. Apart from the reflection of the incident light at the sample surface, the remaining light passing through the composite is attenuated exponentially with material thickness, either through absorption or through scattering at the interface between media with different optical properties, such as the filler and the organic matrix. To enhance light transmission at depth, a reduction in the interface between filler and matrix is sought, either by enlarging the filler size or/and by reducing the amount of filler, along with reducing the amount of pigments and opacifier. In addition, most composites are tuned to become increasingly translucent during polymerization [28], allowing enhanced light transmission in depth upon exposure to light. The increase in translucency upon curing is partly due to the consumption of the photo-initiator, but is mainly achieved by optimizing the refractive index mismatch between filler and resin [29] so that the forming polymer approaches the refractive index of the inorganic fillers. This mechanism is representative of SDR+, which was found to be the most translucent material in the study. Compared to the other composites tested, the light transmittance takes longer to level out in a plateau, but this variation and the time of leveling out correlates well with the kinetic of the C=C double-bond conversion previously observed for the material under similar curing conditions [5].

In contrast to SDR+, TPF experiences completely different kinetic of light transmittance, starting with an sharp and rapid (0.5 s) increase in light transmittance at the highest measured initial rate, then becoming progressively more opaque, rather than increasingly translucent. The initial rapid change in light transmittance is well related to the photo-initiator used, as the material employs a tailored germanium-based photo-initiator (Ivocerin [26]), which is a Norrish type I initiator capable of generating two radicals through alpha cleavage when exposed to light, both of which are able to initiate the polymerization [26]. In addition, the reactivity of the germanium-based photo-initiator is considered to be higher compared to camphorquinone (CQ), due to its higher molar extinction coefficient [26], which allows for a faster and more efficient C=C double-bond conversion. However, it is important to note that a blue LED LCU was used for polymerization, while the germanium-based photo-initiator would benefit from additional violet light, since its absorption maxima is at 411–418 nm, while CQ (λ_max_ = 468 nm) is best adapted to blue light [26]. The steeper change in light transmittance, in turn, correlates well with the real-time C=C double-bond conversion, which was shown to be steeper in TPF compared to SDR+ [5]. The subsequent observed decrease in translucency is a tailored property, designed to match the translucency of the material to that of the tooth after curing, whereas in the uncured state the material differs from the tooth in this respect, in order to visualize proper application. Compared again with SDR+, the refractive index mismatch between filler and resin increases during polymerization, thus resulting in more light scattering at the interface and lower light transmittance, which, however, does not affect the degree of conversion or the mechanical properties [5]. Additionally, in the same comparison, the time it takes to reach a plateau in light transmittance is consistently shorter. The light transmittance behavior therefore no longer correlates with the C=C double-bond conversion, which continues to increase long after no changes in light transmittance are observed [5].

BBFF was the material with less variation in light transmittance during curing and the most opaque one, which can be a result of the relatively high amount of pre-polymerized fillers, as observed in the SEM images. It is promoted as a multi-shade, flowable, bulk-fill composite, but also as a composite that does not need a top layer of highly filled composite materials. The higher opacity was certainly a deliberate development decision, particularly since the composite can be used without capping, as highly translucent materials placed in large cavities may not adequately mask tooth discoloration or may appear grayish. BBFF was recently released, and information about its behavior is scarce [5]. The evolution of light transmittance during light exposure does not correlate with the C=C double-bond conversion measured under similar conditions, as the latter was shown to overlap well with the data measured for SDR+ up to 7 s of light exposure and, beyond that, to flatten out later, as observed in SDR+. This difference in behavior may be related to the very large urethane monomers in SDR+ used to reduce shrinkage stress, resulting in a correspondingly faster restriction of monomer mobility. In the BBFF, the chemical composition of the organic matrix is stated ambiguously as methacrylate-based, which does not allow any further interpretation.

In terms of mechanical behavior, the studied materials present some differences that are well related to their filler content (Table 1). SDR+ and TPF have an almost similar volumetric filler amount, resulting in similar hardness and indentation modulus, while the lower filler amount in BBFF significantly lowers these parameters at 24 h post-polymerization. Creep, the time-dependent deformation measured in the present experiment as a result of an indentation lasting 5 s at a maximum load of 1 N, was highest in SDR+. This result can be attributed to the chemical composition of the organic matrix, as the very large urethane monomers are associated with little cross-linking after polymerization, allowing greater movement of individual chains, thus increasing creep.

The harsh conditions faced by composite restorations in the oral cavity result in premature degradation that shortens their lifespan [30], while hydrolytic degradation remains one of the more difficult challenges [17]. The latter is not limited to the polymer matrix, but also includes the degree of adhesion of the inorganic filler to the organic polymer [31]. In fact, after 6 months of saliva storage at 37 °C, aging affected all materials and resulted in a clear distinction between them, as SDR+ showed a more pronounced decrease, followed by TPF and then BBFF. This changed the ranking of the materials in terms of the measured properties. BBFF still exhibits the lowest hardness and indentation modulus, due to its lower inorganic filler content, but the differences between the materials steadily decreased. This aging behavior is partly unexpected, as materials with pre-polymerized fillers (TPF and BBFF) are expected to degrade more quickly than those with compact fillers (SDR+), with the connection of the pre-polymerized fillers in the organic matrix considered to be the weakest link in a composite [32]. Along the same line of reasoning, urethane methacrylates, such as those contained in SDR+, are expected to induce improved resistance to degradation due to lower water absorption [33,34]. In contrast, the low cross-linking of the very large monomers in SDR+, supported by the creep data, could outweigh the above advantages and exert a greater impact with aging.

Therefore, the tested null hypotheses that modern flowable bulk-fill composite formulation behaves similarly with respect to the kinetic of light transmission during setting, their mechanical parameters, and aging behavior, are all rejected.

## 5. Conclusions

The kinetic of light transmission differ substantially between the materials analyzed due to differences in the chemical composition of the materials, including the degree of mismatch between the refractive indices of filler and polymer matrices during polymerization or/and the type of initiator used. The kinetic of light transmission do not generally correlates with the kinetic of functional group conversion. Therefore, light transmittance should not be used to assess cure quality or to determine exposure time. Furthermore, the initial mechanical properties are directly related to the volumetric amount of filler, but degradation must be considered as a multifactorial event.

## Figures and Tables

**Table 1:** Analyzed flowable bulk-fill composites: material abbreviation (code), brand, manufacturer, LOT and filler amount, as indicated by the manufacturer. Exposure time was 20 s in all materials (n.s. = not specified, wt.% = weight percent; vol.% = volume percent).

Code	Brand	Manufacturer	LOT	Filler wt./vol.%
BBFF	Brilliant Bulk Fill Flow	Coltene	M38595	56.0/38.5
SDR+	SDR flow+ universal	Dentsply Sirona	2206000502	n.s./47.3
TPF	Tetric Power Flow, ^IV^ A	Ivoclar Vivadent	Z03W68	68.2/46.4

**Table 2:** Parameters of the exponential sum function used to fit the real-time variation of the transmitted irradiance as a function of exposure time for the three analyzed materials. Coefficient of determination (R^2^) and the standard error (SE) for the calculated parameters are given.

Cod	R^2^	a, SE		b, SE		c, SE		d, SE	
BBFF	0.96	353.4	0.51	40.33	2.37	23.27	4.3	1.37	0.001
SDR+	0.99	475.5	0.42	50.27	0.96	145.9	0.39	0.33	0.001
TPF	0.98	489.2	2.94	23.03	0.56	−16.85	2.92	1.15	0.24

**Table 3:** Multivariate analysis (general linear model) and effect strength on the measured parameters expressed by the partial eta-squared values of the main parameters—composite and filler volume%—as well as their binary interaction.

Parameter	HV, N/mm^2^	HM, N/mm^2^	E_IT_/(1 − vs^2^), GPa	Cr, %
Aging	0.72	0.74	0.83	0.54
Vol. %	0.91	0.93	0.96	0.45
Aging × Vol. %	0.56	0.54	0.61	0.46

## Data Availability

The raw data supporting the conclusions of this article will be made available by the authors on request.
